# Activities of (Poly)phenolic Antioxidants and Other Natural Autophagy Modulators in the Treatment of Sanfilippo Disease: Remarkable Efficacy of Resveratrol in Cellular and Animal Models

**DOI:** 10.1007/s13311-022-01323-7

**Published:** 2022-11-07

**Authors:** Estera Rintz, Magdalena Podlacha, Zuzanna Cyske, Karolina Pierzynowska, Grzegorz Węgrzyn, Lidia Gaffke

**Affiliations:** Department of Molecular Biology, Faculty of Biology, University of Gdansk, Wita Stwosza 59, 80-308 Gdansk, Poland

**Keywords:** Autophagy inducers, Resveratrol, Sanfilippo disease, Novel therapy

## Abstract

Sanfilippo disease, caused by mutations in the genes encoding heparan sulfate (HS) (a glycosaminoglycan; GAG) degradation enzymes, is a mucopolysaccharidosis (MPS), which is also known as MPS type III, and is characterized by subtypes A, B, C, and D, depending on identity of the dysfunctional enzyme. The lack of activity or low residual activity of an HS-degrading enzyme leads to excess HS in the cells, impairing the functions of different types of cells, including neurons. The disease usually leads to serious psychomotor dysfunction and death before adulthood. In this work, we show that the use of molecules known as dietary (poly)phenolic antioxidants and other natural compounds known as autophagy activators (genistein, capsaicin, curcumin, resveratrol, trehalose, and calcitriol) leads to accelerated degradation of accumulated HS in the fibroblasts of all subtypes of MPS III. Both the cytotoxicity tests we performed and the available literature data indicated that the use of selected autophagy inducers was safe. Since it showed the highest effectivity in cellular models, resveratrol efficacy was tested in experiments with a mouse model of MPS IIIB. Urinary GAG levels were normalized in MPS IIIB mice treated with 50 mg/kg/day resveratrol for 12 weeks or longer. Behavioral tests indicated complete correction of hyperactivity and anxiety in these animals. Biochemical analyses indicated that administration of resveratrol caused autophagy stimulation through an mTOR-independent pathway in the brains and livers of the MPS IIIB mice. These results indicate the potential use of resveratrol (and possibly other autophagy stimulators) in the treatment of Sanfilippo disease.

**Supplementary Information:**

The online version contains supplementary material available at 10.1007/s13311-022-01323-7.

## Introduction

**Fig. 1:**
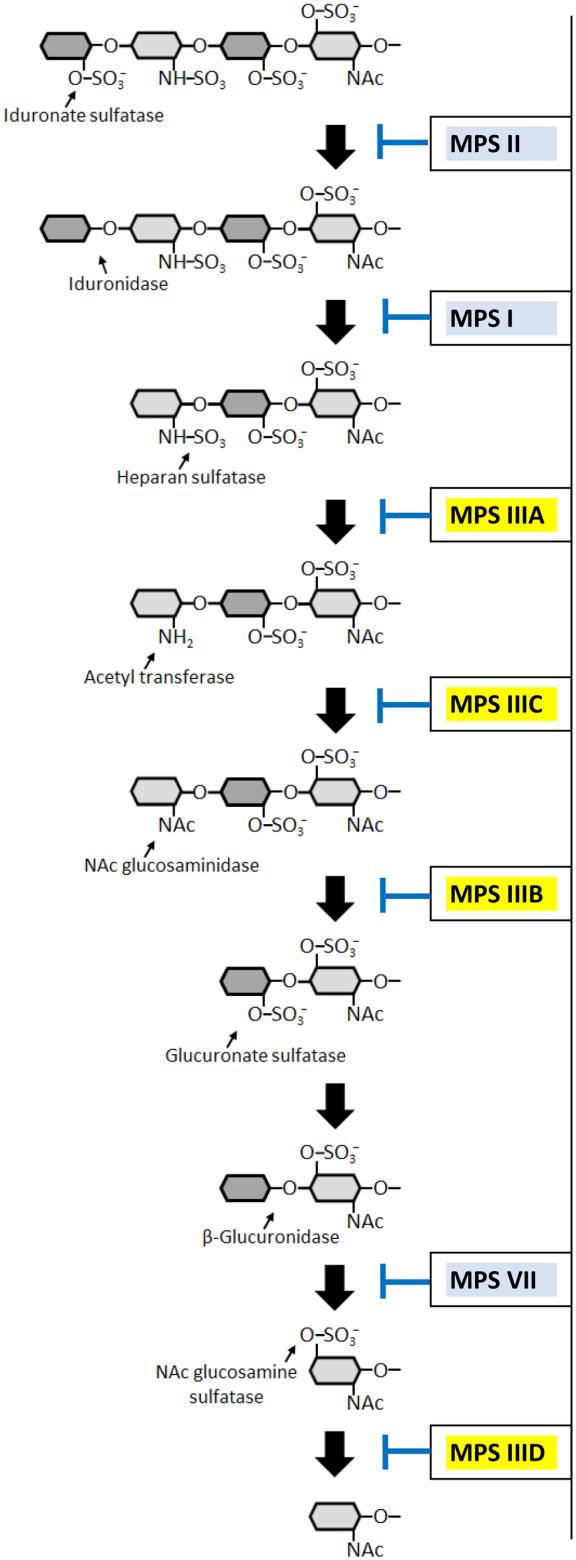
The pathway of heparan sulfate (HS) degradation. The HS molecule consists of repeated polysaccharide fragments composed of the following mono-sugars: 2-O-sulfo-α-L-iduronic acid, 2-deoxy-2-sulfamido-α-D-glucopyranosyl, 2-O-sulfo-glucuronic acid, and 2-deoxy-2-acetamido-α-D-glucopyranosyl sulfate. Enzymes involved in the sequential steps of HS degradation are indicated with arrows showing the specific reactions they catalyze. As a result of deficiency in any one of these enzymes, the degradation process is halted at a specific stage (symbolized by blunt-ended lines), causing the accumulation of undegraded HS-derivative and the development of symptoms corresponding to an MPS type/subtype (marked in boxes). The disease subtypes with accumulated HS as the only primary molecule stored in excess are commonly grouped into the MPS III (Sanfilippo disease) A, B, C, and D subtypes (marked in yellow), based on ref. [1] with modifications

Sanfilippo disease (mucopolysaccharidosis type III or MPS III) belongs to a group of lysosomal storage diseases (LSDs) caused by mutations leading to the accumulation of a glycosaminoglycan (GAG): heparan sulfate (HS) [1]. In MPS III, one of the following genes encoding HS-degrading enzymes is pathologically mutated: the *SGSH* gene (encoding N-sulfatase heparan), *NAGLU* gene (encoding α-N-acetylglucosaminidase), *HGSNAT* gene (encoding acetyl-CoA:α-glucosaminide acetyltransferase), and *GNS* gene (encoding N-acetylglucosamine-6-sulfatase), resulting in one of the 4 subtypes of the disease, MPS III A, B, C, and D, respectively [2]. The pathways of HS degradation, enzymes involved in this process, and MPS types/subtypes caused by deficiencies of specific enzymes are presented in Fig. 1.

MPS III is a neuronopathic mucopolysaccharidosis [3]. Patients affected by this disease present with a set of common traits that include developmental delay, sleep disturbances, aggressive behavior, cognitive decline, hyperactivity, and seizures [4]. These symptoms sometimes appear at the age of a few months, but they are usually evident when an affected child is a few years old. Because these symptoms are not unique to MPS III, this disease is often misdiagnosed in its early stages [5]. The expected lifespan of Sanfilippo disease patients is approximately 2 decades [1].

Despite various attempts to develop a therapy for Sanfilippo disease, no treatment has been shown to be effective in patients to date [6]. In fact, although many encouraging results of various therapeutic approaches have been reported in preclinical trials (including enzyme replacement therapy, gene therapy, substrate reduction therapy, and others [7–9]), no therapy has been approved for MPS III [10]. Severe neurodegeneration is a major obstacle in the development of an effective therapy for this disease, as it is challenging to find an efficient way to deliver potential drugs, especially large molecules, to the brain [6]. Moreover, recent studies have indicated that although HS storage is the major pathomechanism of the disease, secondary processes, including the dysregulated expression of hundreds of genes encoding proteins involved in the structures and functions of various cellular organelles, as well as different cellular processes (such as metabolic processes, cell communication, signal transduction, cell development, movement of subcellular components, and the cell cycle), may significantly influence the pathomechanisms of Sanfilippo disease, hindering the development of effective therapies [11–14]. Recent failures in the clinical improvements of MPS III patients participating in clinical trials with different kinds of therapies, such as gene therapy [15], enzyme replacement therapy [16, 17], and substrate reduction therapy [18], have confirmed the great challenge to finding an effective therapy for Sanfilippo disease [19].

In this light, novel therapeutic approaches to MPS III are highly desirable. Autophagy is a highly conserved process in all eukaryotic cells, functioning at a low level and activated largely by the action of various external factors or by incorrectly folded macromolecules. A macromolecule destined for degradation is encompassed by an insulating membrane, forming a vesicle called an autophagosome. An autophagosome fuses with a lysosome, and lysosomal enzymes digest the macromolecules in the autophagolysosome, forming single monomers that can be reused by the cell [20]. Induction of autophagy has been proposed as a promising therapeutic strategy for neurodegenerative diseases associated with protein aggregation (such as Huntington’s disease, Alzheimer’s disease, and Parkinson’s disease), as stimulated removal of these toxic structures might restore cellular homeostasis [21]. However, autophagy has rarely been evaluated as an MPS III treatment strategy, although suggestions about its possible effectiveness have been published [22–26]. However, autophagy is not limited to the degradation of proteins; in fact, it is a poorly selective process and can remove stored carbohydrates such as GAG. Therefore, the broad spectrum of neurodegenerative diseases might be considered when assessing autophagy-related treatments. Nevertheless, any potential pharmacological treatment of a genetic disease is necessarily a long-term strategy; therefore, a putative drug must be safe for long-term use. Unfortunately, most strong exogenous autophagy activators are deleterious to cells when used for a long time; therefore, they are not appropriate candidates for treatments against genetic diseases [27]. Hence, in our studies, we have focused on natural compounds that are safe for patients to use long-term and that effectively stimulate autophagy [28].

In this study, we asked whether pharmacological stimulation of autophagy by naturally occurring molecules can accelerate the degradation of stored GAG in MPS III. In this work, we tested the following known phenolic compounds and other natural autophagy stimulators: capsaicin [29, 30], curcumin [31–33], resveratrol [34, 35], trehalose (the only autophagy stimulator previously reported to be a potential drug for MPS III treatment) [22, 36, 37], and calcitriol [38, 39]. Since resveratrol showed the greatest capacity to reduce the amount of stored GAG (HS) in all subtypes of MPS III, as demonstrated in experiments with fibroblast cultures, this compound was chosen for further study with a mouse model of MPS IIIB. These experiments allowed us to test a recently proposed hypothesis suggesting that resveratrol might be a potential therapeutic agent in the treatment of MPS [40]. Our results confirmed the high potential of natural autophagy inducers, especially resveratrol, in the treatment of Sanfilippo disease, as assessed in experiments with cell and animal models.

## Methods

### Cell Cultures

Lines of fibroblasts derived from patients suffering from Sanfilippo disease types A, B, C, and D, as well as a control cell line of human fibroblasts (the HDFa line), were assessed. MPS III fibroblasts were purchased from the NIGMS Human Genetic Cell Repository at the Coriell Institute for Medical Research (this Institute provides all documentation related to bioethical issues). The following cell lines were used: HDFa — control fibroblasts derived from a healthy person; MPS IIIA — fibroblasts (NIGMS Cat No. GM00879) from a Caucasian, 3-year-old, female patient with mutations in the *SGSH* gene: c.G1351A/G746A (p.Glu447Lys/p.Arg245His); MPS IIIB — fibroblasts (NIGMS Cat No. GM00156) from a 7-year-old Caucasian male patient with mutations in the *NAGLU* gene: c.C1876T/C1876T (p.Arg626Ter/p.Arg626Ter); MPS IIIC — fibroblasts (NIGMS Cat No. GM05157) from an 8-year-old male patient of unknown race with mutations in the *HGSNAT* gene and diagnosed on the basis of estimated urinary GAG levels and the activity of the corresponding enzyme in plasma; MPS IIID — fibroblasts (NIGMS Cat No. GM05093) from a 7-year-old Asian–Indian male patient with mutations in the *GNS* gene: c.C1063T/C1063T (p.Arg355Ter/p.Arg355Ter). Cells (passages 6–17) were cultured in flasks with DMEM (Thermo Fisher Scientific Inc., Paisley, UK) supplemented with 10% FBS (Thermo Fisher Scientific Inc., Paisley, UK) and a 1% antibiotic/antimycotic solution (Sigma‒Aldrich Co. LLC., St. Louis, USA) at 37 °C in a humidified atmosphere with 5% CO_2_.

### Reagents

Genistein (99% purity; #446–72-0) was purchased from the Pharmaceutical Research Institute in Warsaw (Poland). It was dissolved in DMSO to obtain stock solutions of 30, 60, and 100 mM; these solutions were stored at −20 °C. Capsaicin (≥ 99% purity; #M2028) was purchased from Sigma‒Aldrich, USA. It was dissolved in DMSO to obtain stock solutions of 10, 50, or 100 mM; these solutions were stored at −20 °C. Curcumin (Cay81025) was purchased from Cayman Chemical, USA. It was dissolved in DMSO to obtain stock solutions of 10, 30, and 80 mM; these solutions were stored at −20 °C. Resveratrol (#T1558) was purchased from TargetMol (USA). It was dissolved in DMSO to obtain stock solutions of 20, 80, and 160 mM; these solutions were prepared and stored at −20 °C. Trehalose (Cay20517) was purchased from Cayman Chemical, USA. It was dissolved in distilled water to obtain a stock solution of 1 M; this solution was stored at −20 °C. Calcitriol (#C0225000) was purchased from Sigma‒Aldrich, USA. It was dissolved in 96% ethanol to obtain stock solutions of 10, 50, and 100 mM; these solutions were stored at −20 °C. Thiazolyl blue tetrazolium bromide (98% purity; #M2128) was purchased from Sigma‒Aldrich. MTT was dissolved in PBS (4 mg/ml) and stored at 4 °C.

### Cell Viability Assay

Cells (3 × 10^3^) were seeded in the wells of 96-well plates and allowed to attach overnight. They were then treated with either DMSO, distilled water or 96% ethanol (control cells) or increasing concentrations of the test compound for 24 h. Then, 25 µl of MTT (3-(4,5-dimethylthiazol-2-yl)-2,5-diphenyltetrazolium bromide) solution (4 mg/ml) was added to each well. Following a 3-h incubation at 37 °C, formazan crystals that formed in living cells were dissolved in 100 µl of DMSO. Absorbance was measured at 570 nm and 660 nm (the reference wavelengths) with a Victor3 microplate reader.

### Measurement of Glycosaminoglycan (GAG) Levels in Fibroblasts

For the determination of GAG levels in fibroblasts, 1 × 10^5^ cells were seeded in each well of a 6-well plate, and they were allowed to attach overnight. The cells were then treated with either DMSO, distilled water, or 96% ethanol (control cells) or increasing concentrations of the test compound for 24 h and collected in tubes via trypsinization. The level of GAG in the cells was measured using a Glycosaminoglycan Assay Blyscan kit (Biocolor Ltd., Carrickfergus, UK) following the manufacturer’s instructions.

Levels of heparan sulfate (HS) in fibroblasts were estimated using extracts from cells treated with lysis buffer (1% Triton X-100, 0.5 mM EDTA, 150 mM NaCl, and 50 mM Tris, pH 7.5) and the dot–blot procedure with a PVDF membrane (IPFL00010, Millipore). Briefly, cell extracts were fixed to the membrane using a dot–blot apparatus (Bio–Rad, Hercules, CA, USA). The membrane was blocked with 5% nonfat dry milk in PBST buffer and incubated with a primary mouse anti-HS antibody (NBP-2–23,523, Novus) overnight at 4 °C. Then, the membrane was washed with PBST and incubated with a secondary anti-mouse antibody coupled with peroxidase (#A9044, Sigma Aldrich, USA) for 1 h at room temperature. Following treatment with a chemiluminescent HRP substrate (Merck, Darmstadt, Germany) solution, the membrane was exposed to X-ray film. The intensities of the dots were analyzed with QuantityOne software. The values were normalized to the total protein amount as determined by Ponceaus S staining.

### Animals

The B6.129S6-Naglu^tm1Efn^/J mouse model of MPS IIIB (*Naglu*^−/−^) was used (JAX stock #003,827, purchased from Jackson Laboratories (Sacramento, CA, USA)). MPS IIIB mice were backcrossed with C57BL/6 J (WT) mice. Each group of mice consisted of six males (*n* = 6). This size of each group was calculated via power analysis, which indicates the number of animals (*n* = 6) to include in each experimental group to achieve an *α* = 5% as the significance level for a statistical test with a power of 95%. All animal procedures were approved by the Local Ethics Committee for Animal Experiments in Bydgoszcz (application approval no. BUD13/2020) and carried out according to the guidelines of the European Communities Council Directive (2010/63/UE). The mice were housed with a 12/12-h light/dark cycle and with food and water provided ad libitum.

### Administration of Resveratrol or Water to Mice

Resveratrol was administered to mice by an orogastric probe once per day. The dose was 50 mg/kg mouse weight/day, and the duration of the treatment was 22 weeks (starting when the mice were 8 weeks old). Stock volumes of resveratrol suspension in water were prepared in such a way that a single dose administered to a mouse was 0.1 ml or lower. The control group of mice received 0.1 ml of distilled water administered by orogastric probe, exactly as resveratrol treatment of the mice, every day.

### Determination of Urinary GAG Levels

Urine samples were collected using commercially available hydrophobic sand (LabSand, Coastline Global, Palo Alto, CA) at four time points: when the mice reached 5, 10, 20, and 30 weeks of age. The material was collected at a fixed time between 10:00 a.m. and 12:00 a.m. This method is much less stressful for the animals than the use of metabolic cages, and it reduces the influence of additional factors that can interfere with the evaluation of the parameters of interest. In brief, a portion of hydrophobic sand was poured into the bottom of a cage measuring 15.2 cm × 25.4 cm with a surface area of 386 cm^2^. The mice were individually placed in the cage and prepared for testing. Prior to urine sampling, the animals had no access to water or food (for 2 h), but their movement was not restricted in any way. Due to the special properties of the sand used, the urine samples stayed on the surface and were easily collected with a pipette. Samples of mouse urine were prepared for measurements by making serial dilutions (with a final sample volume of 0.1 ml) to obtain values that could be compared to a calibration curve obtained using known GAG concentrations. GAG levels were measured using a commercial kit (a BlyscanTM Glycosaminoglycan Assay, Biocolor Ltd., Carrickfergus, UK).

### Behavioral Tests

The locomotion of the mice was measured with an actometer. Experiments were performed as described previously [41]. The numbers of horizontal, vertical, and ambulatory movements within a time period were measured.

Anxiety-related locomotion activity was investigated in an open field. Previously described experimental procedures were employed [41]. The following parameters were measured for each investigated mouse: freezing and exploratory activity; the total amount of time spent in inner/outer squares; the total number of inner/outer squares entered by the animal; the number of lines crossed; and the frequency of mouse entry with all four paws in an inner/outer arena.

All behavioral analyses were recorded using EthoVision XT 10 software (Noldus, Wageingen, the Netherlands). However, the results were not solely based on the automated analysis performed with this software, as each video was additionally reviewed by the experimenter to detect atypical behavior. Despite the fact that certain types of reactions seem contradictory, the observations indicated that the MPS IIIB mice exhibited hyperactivity that was very chaotic in nature, as these behaviors were interrupted with episodes of immobility and other deviations from the pattern observed in the treated or control mice.

### Determination of Protein Levels by Western Blotting

Relative levels of selected proteins in the brains and livers of mice were measured by Western blot experiments using a WES system (WES — Automated Western blots with Simple Western; ProteinSimple, San Jose, CA, USA). Mice were euthanized at 30 weeks of age with Morbital (2 ml/kg). After separation from organs, tissue samples were homogenized in IG buffer (0.9% NaCl, 0.5% Triton X-100, 0.1% SDS, 1% sodium deoxycholate, 5 mM EDTA, and 50 mM Tris–HCl pH 7.5) and prepared as described previously [41]. Proteins were separated in a WES system using the 12–230 kDa separation module with 8 × 25 capillary cartridges (#SM-W004; ProteinSimple, San Jose, CA, USA). Specific proteins were detected with the following antibodies: anti-SQSTM1/P62 mouse antibody (#sc-48402, Santa Cruz Biotechnology, Santa Cruz, CA, USA), anti-Beclin-1 rabbit antibody (#D40C5, Cell Signaling Technology, Danvers, MA, USA), anti-PI3 kinase class III rabbit antibody (#D9A5, Cell Signaling Technology, Danvers, MA, USA), anti-TFEB rabbit antibody (#D207D, Cell Signaling Technology, Danvers, MA, USA), anti-S6K rabbit antibody (#9202; Cell Signaling Technology, Danvers, MA, USA), anti-phospho-p70 S6 kinase (Thr389) anti-rabbit antibody (#9205, Cell Signaling Technology, Danvers, MA, USA), anti-4ebp1 rabbit antibody (#9452, Cell Signaling Technology, Danvers, MA, USA), and anti-phospho-4E-BP1 (Thr37/46) rabbit antibody (#9459, Cell Signaling Technology, Danvers, MA, USA). For the detection procedure, secondary antibodies (either anti-mouse or anti-rabbit), which were included in an anti-mouse detection module (#DM-002, ProteinSimple, San Jose, CA, USA) or an anti-rabbit detection module (#DM-001, ProteinSimple, San Jose, CA, USA), were used. The total protein level, determined using a total protein detection module for chemiluminescence (#DM-TP01, ProteinSimple, San Jose, CA, USA), was used as the loading control. Quantification of the results was performed using the software included in the WES system.

For the measurement of LAMP-2 and LC3-II levels, the classical Western blot procedure was employed using an anti-LAMP-2 mouse antibody (H4B4, #sc-18822, Santa Cruz Biotechnology, Santa Cruz, CA, USA) and anti-LC3 mouse antibody (G-4, #sc-398822, Santa Cruz Biotechnology, Santa Cruz, CA, USA). In these experiments, the GAPDH protein level, as measured with an anti-GAPDH rabbit antibody (14C10, #2118 Cell Signaling Technology, Danvers, MA, USA), was used as the control. The performance of classical Western blotting was necessary because certain proteins, including the LC3-II form, are not effectively separated with the WES system, as indicated in the guidelines for studies on autophagy [42]. The results were quantified via densitometry.

### Statistical Analysis

The values are presented as the mean ± SD or SEM. For statistical analysis of the results, SPSS 21.0 (SPSS Inc., Amonk, USA) software was used. All parameters for in vitro experiments were analyzed via two-way ANOVA and Tukey’s post hoc test or the Kruskal‒Wallis and Dunn test. The choice of a test was dictated by the fulfillment of two basic assumptions indicating the appropriate use of a parametric analysis. When the distribution of the results was normal and the variances were homogeneous, a two-way ANOVA was performed. When on or both one of these assumptions were not met, a nonparametric analysis was performed. The differences were considered statistically significant when *p* < 0.05.

## Results

### Toxicity of the Tested Compounds to MPS III Fibroblasts

We aimed to test the effectivity of select dietary (poly)phenolic antioxidants and certain other natural compounds known to activate autophagy in the treatment of cells derived from patients suffering from all subtypes of Sanfilippo disease (MPS III). In the first step, we tested whether these compounds induced cytotoxicity in the MPS fibroblasts.

**Fig. 2:**
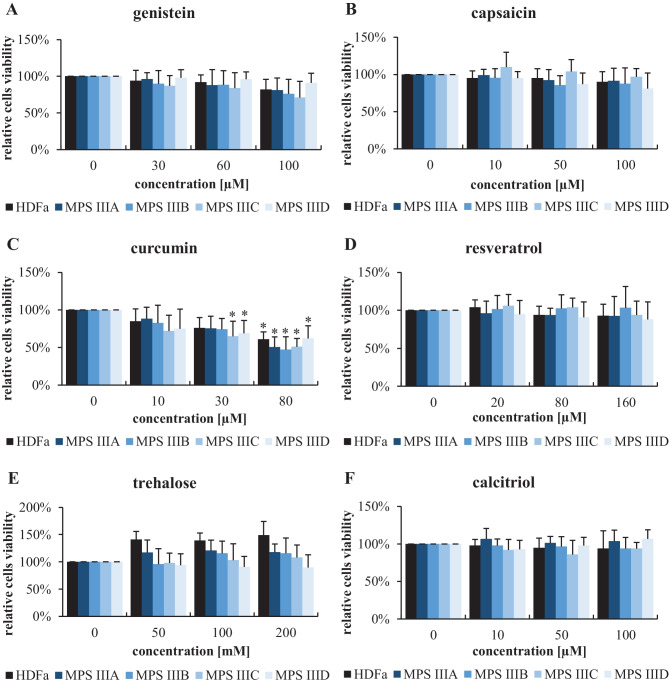
Viability of control (HDFa) and MPS III (subtypes A-D) fibroblasts treated with various compounds at the indicated concentrations for 24 h, as estimated by MTT test. The results obtained at time 0 were considered to indicate 100% viability. Each column represents the mean value of three independent experiments, and error bars indicate the standard deviation. Asterisks indicate significant differences (*p* < 0.05) between the results at time 0 and other time points

An MTT assay was used in experiments with MPS IIIA, IIIB, IIIC, and IIID fibroblasts and all the tested compounds: genistein (used as a positive control, which had been previously demonstrated to decrease the levels of GAG by acting as both a negative regulator of the synthesis of these compounds and an autophagy stimulator), capsaicin, curcumin, resveratrol, trehalose, and calcitriol. The concentration ranges of all tested compounds were chosen on the basis of previously published results, indicating that, at these levels, these treatments stimulate autophagy efficiently [29–39]. We found that most of these molecules did not affect the viability of the MPS III cell lines or the HDFa cell controls, when used in the concentration ranges previously reported as standards for in vitro experiments (Fig. 2). A significant decrease in cell viability was found only in the presence of curcumin at 30 and 80 μM (Fig. 2).

### Reduction in GAG Levels by the Tested Compounds

**Fig. 3:**
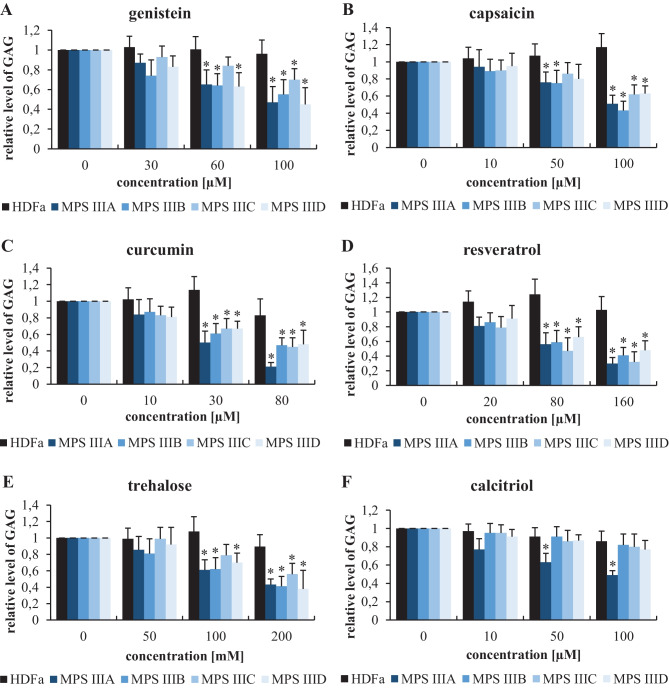
Relative GAG levels in control (HDFa) and MPS fibroblasts treated with various compounds at the indicated concentrations for 24 h, as assessed by the Blyscan assay. The results obtained at time 0 were considered to be 1 for each separate cell line. Each column represents the mean value of three independent experiments, and error bars indicate the standard deviation. Asterisks indicate significant differences (*p* < 0.05) between the results at time 0 and other time points

Since deficiency in the activity of one of the enzymes involved in GAG degradation is a primary cause of an MPS, we evaluated whether treatment of MPS III cells with the tested compounds results in the reduced accumulation of GAG. Concentrations of GAG were determined by performing Blyscan assays, which indicated any increase in the amounts of GAG in MPS III cells relative to those in the HDFa control cells (when compared to the control cell line, the GAG levels were increased 2.3-, 3.2-, 4.1-, and 2.2-fold in the MPS IIIA, IIIB, IIIC, and IIID fibroblasts, respectively). Genistein has been used as a positive control for the tested molecules, which was previously demonstrated to cause a reduction in GAG levels in MPS cells due to its activity as an indirect inhibitor of GAG synthesis and stimulator of lysosomal biogenesis [43–47]. As expected, when applied at concentrations of 60 or 100 μM for 48 h, this natural polyphenol (acting as an antioxidant) caused a significant decrease in GAG levels in all subtypes of MPS III (Fig. 3). Interestingly, similar effects were observed for all the tested compounds, although the reduction in GAG levels was especially pronounced in experiments with curcumin, trehalose, and resveratrol, and was less pronounced with calcitriol (Fig. 3). However, since curcumin showed some negative effects on cell viability (see Fig. 2) and because the effects of trehalose on MPS IIIB mice had been reported previously [22], resveratrol was chosen for further and more detailed studies.

### Resveratrol-Mediated Reduction of HS Levels in MPS III Cells

**Fig. 4:**
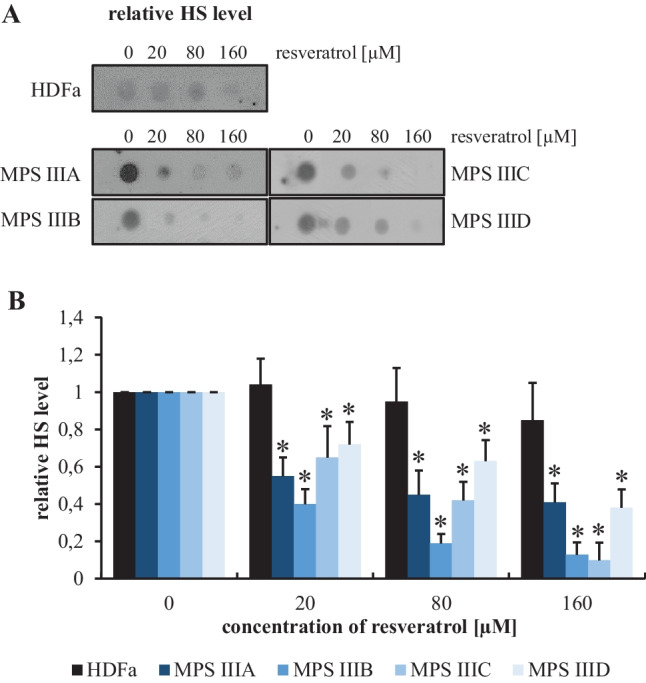
HS levels in control (HDFa) and MPS fibroblasts treated with resveratrol at the indicated concentrations for 24 h, as assessed by the dot–blot assay with a specific anti-HS antibody. Panel **A** indicates representative results, while panel **B** shows the quantitation of the results in (**A**). In panel **B**, the results obtained at time 0 were considered to be 1 for each separate cell line. Each column represents the mean value of three independent experiments, and error bars indicate the standard deviation. Asterisks indicate significant differences (*p* < 0.05) between the results at time 0 and other time points

Since HS is the primary GAG stored in MPS III (secondary dermatan sulfate storage has also been reported in Sanfilippo disease [48]), we have tested the efficiency of resveratrol in the reduction of HS levels in fibroblasts derived from patients suffering from all subtypes of MPS III. A specific anti-HS antibody was employed, and the concentrations of HS d in the extracts of fibroblasts were estimate using a dot–blot procedure. When assessing basic levels of HS in untreated fibroblasts, the concentrations of GAG were increased by factors of 4.8, 3.4, 3.7, and 3.2 in MPS IIIA, IIIB, IIIC, and IIID, respectively, relative to control cells (HDFa) (Fig. 3A). Treatment with resveratrol at concentrations of 20, 80, and 160 μM for 48 h resulted in significant decreases in HS levels in all subtypes of Sanfilippo disease cells, and a dose‒response correlation was observed (Fig. 4B). These results indicated that resveratrol was effective in clearing HS from MPS III cells in vitro, pointing to its therapeutic potential in the treatment of Sanfilippo disease. Therefore, in the next step, we tested the efficacy of resveratrol in an experimental therapy of mouse models of MPS IIIB.

### Resveratrol-Mediated Reduction in Urinary GAG Levels in MPS IIIB Mice

**Fig. 5:**
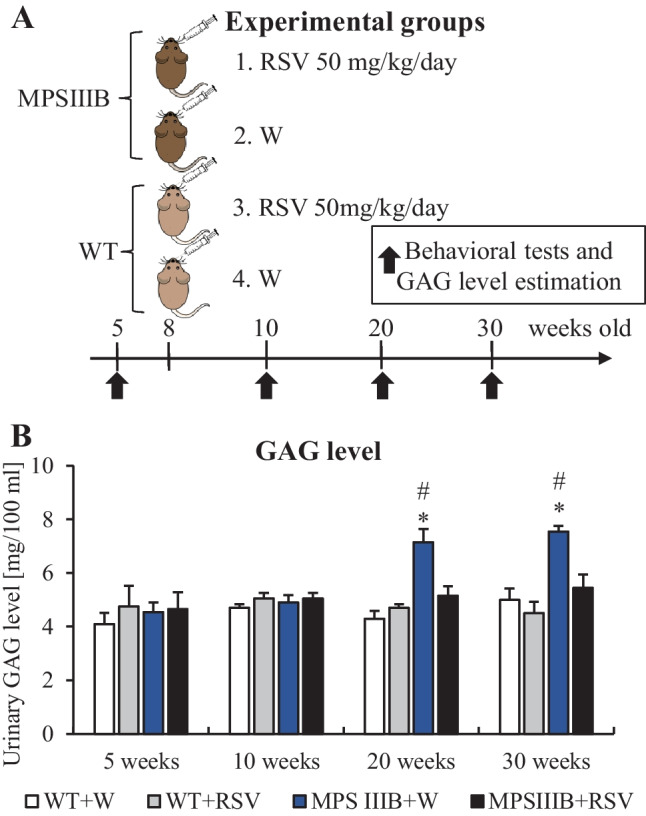
Scheme of the animal experiments (**A**) and effects of resveratrol (RSV) on urinary GAG levels in MPS IIIB mice (**B**). The results shown in panel **B** are presented as the mean values ± SEMs. Statistically significant differences obtained for a particular group (*n* = 6) are marked with (*), which indicates *p* < 0.05 vs. WT + W (wild type with water), or (#), which indicates *p* < 0.05 vs. MPS IIIB + RSV (MPS IIIB treated with resveratrol)

To test the effects of resveratrol on the course of Sanfilippo disease, we used a mouse model of MPS IIIB. The animals were allocated into 4 groups: wild-type (WT; *Naglu*^+/+^) mice treated with water (W), wild-type mice treated with resveratrol (RSV; 50 mg/kg/day), MPS IIIB (*Naglu*^−/−^) mice treated with water (W), and MPS IIIB mice treated with resveratrol (RSV; 50 mg/kg/day) (Fig. 5A). The dose of 50 mg/kg/day was chosen as the concentrations of resveratrol on the basis of our in vitro experiments, which caused an effective reduction in GAG levels (as determined by the molecular mass of resveratrol, which is equal to 228.25 g/mol, and assuming ~50% bioavailability of the orally administered compound). Both water and resveratrol were administered orally. The treatment was initiated in mice 8 weeks old and was conducted until the mice were 30 weeks old. Measurements of the levels of urinary GAG and behavioral tests were performed at weeks 5, 10, 20, and 30 (Fig. 5A).

Levels of GAG in urine were found to be similar in both WT and MPS IIIB mice at weeks 5 and 10, with or without resveratrol treatment. However, MPS III mice not treated with an investigated compound showed increased urinary GAG concentrations at weeks 20 and 30 (Fig. 5B). Treatment with resveratrol at 50 mg/kg/day normalized the levels of urinary GAG (the GAG concentrations were indistinguishable from those measured in the urine of wild-type mice, as shown in Fig. 5B). These results indicated that this polyphenol was effective, causing a significant reduction in or the complete clearance of urinary GAG in MPS III mice. Therefore, we asked whether this molecule can positively influence the behavior of mouse models of Sanfilippo disease.

### Effects of Resveratrol on the Behavior of MPS IIIB Mice

As behavioral problems are the most pronounced symptoms in Sanfilippo disease [1], we aimed to investigate the effects of resveratrol on the behavior of MPS IIIB mice. Hyperactivity and anxiety-related changes are characteristic of patients suffering from this disease, as well as of corresponding animal models [2–4]; therefore, we performed experiments allowing us to assess the movements and fear-related activities of the mouse models.

**Fig. 6:**
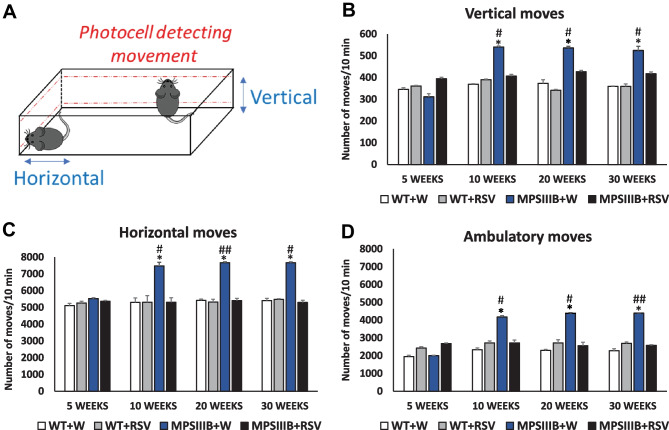
Normalization of hyperactivity in MPS IIIB mice after resveratrol (RSV) treatment. Schematic representation of the actometer used for measuring animal movements via photocell detection is shown in panel (**A**). The numbers of vertical (**B**), horizontal (**C**), and ambulatory (**D**) movements in a 10-min period are presented. The data represent the mean values ± SEMs. Statistically significant differences obtained for each group (*n* = 6) are marked with (*), which indicates *p* < 0.05 vs. WT + W (wild type with water), or (#) and (##), which indicate *p* < 0.05 and *p* < 0.01, respectively, vs. MPS IIIB + RSV (MPS IIIB treated with resveratrol)

Using an actometer, we measured different kinds of movement, including vertical, horizontal, and ambulatory movements (Fig. 6A). All kinds of movements were significantly more frequent in the water-treated MPS IIIB mice than in the WT animals that were 10 weeks older or older (Fig. 6). However, the treatment of mice with Sanfilippo disease B with resveratrol at a dose of 50 mg/kg/day resulted in the complete attenuation of all types of aberrant movements (Fig. 6). Therefore, these results indicated that resveratrol attenuated the hyperactivity in MPS IIIB mice. Moreover, these effects were observed as soon as 2 weeks after the initial administration of this compound (treatment was initiated at the age of 8 weeks, while significant improvement was evident at the age of 10 weeks and later, as demonstrated in Fig. 6).

**Fig. 7:**
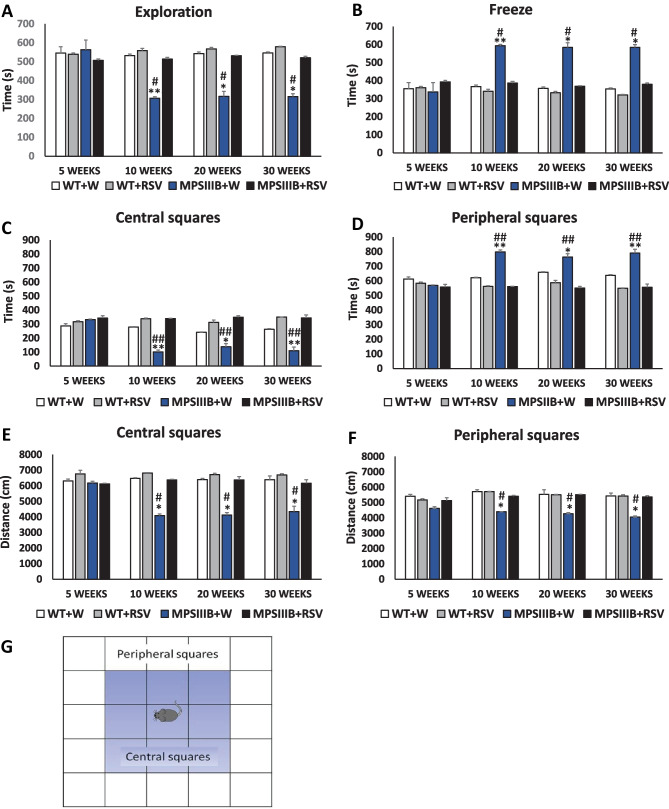
Normalization of anxiety-related behavior in MPS IIIB mice after resveratrol (RSV) treatment. The time spent in exploration (**A**), freezing (**B**), central (**C**), and peripheral (**D)** squares during the 15-min measurement was determined. The distance traveled in central (**E**) and peripheral (**F**) squares over 15 min was measured. A schematic of the open field test is shown in panel (**G**). Data represent the mean values ± SEMs. Statistically significant differences obtained for each group (*n* = 6) are marked with (*) and (**), which indicate *p* < 0.05 and *p* < 0.01, respectively, vs. WT + W (wild type with water), or (#) and (##), which indicate *p* < 0.05 and *p* < 0.01, respectively, vs. MPS IIIB + RSV (MPS IIIB treated with resveratrol)

Performing an open-field test, we estimated fear-related changes in animal behavior. Since anxiety disorders are characteristic of both MPS III (all subtypes) patients and MPS IIIB mice, experiments based on this test were used to measure of these abnormalities. Various parameters were measured to assess animal behavior in the open field, including time of exploration, time of freezing, time spent in central squares, time spent in peripheral squares, distance traveled in central squares, and distance traveled in peripheral squares. The scheme of the experiment is shown in Fig. 7G.

When water-treated animals were investigated, abnormalities in all of the above-listed parameters measured in the open-field test were found in the MPS IIIB mice but not the WT mice (Fig. 7A–F). However, administration of resveratrol normalized all the behavioral functions that had been estimated using this test (Fig. 7). Again, the attenuation of the hyperactive behaviors was evident at the age of 10 weeks, at which time the resveratrol-treated MPS IIIB mice were behaviorally undistinguishable from the WT animals, while the behaviors of water-treated Sanfilippo type B mice were significantly different from those of the control animals for all the measured parameters (Fig. 7). These results demonstrated that resveratrol was a very efficient in correcting behavioral abnormalities in the mouse models of Sanfilippo disease used in this study.

### Resveratrol Induces Autophagy in the Brains and Livers of MPS IIIB Mice

**Fig. 8:**
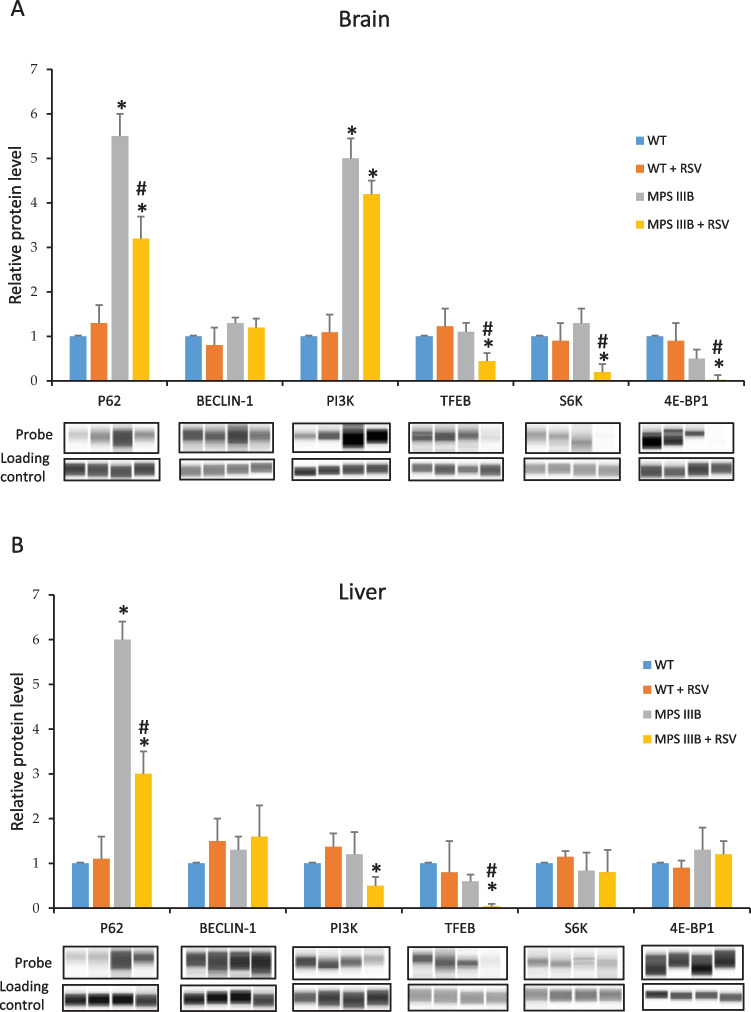
Levels of autophagy-related proteins in the brains (**A**) and livers (**B**) of wild-type mice treated with water (WT) or resveratrol at 50 mg/kg/day (WT + RSV) and MPS IIIB mice (analogous to the MPS IIIB and MPS IIIB + RSV groups). The data represent the mean values ± SDs. Statistically significant differences obtained for each group (*n* = 6) are marked with (*), which indicates *p* < 0.05 vs. WT (wild type with water), or (#), which indicates *p* < 0.05 vs. MPS IIIB + RSV (MPS IIIB treated with resveratrol). Representative blots with the tested proteins (probe) and loading controls (total protein assessed using the total protein detection module for chemiluminescence compared with the WES system) are presented below the corresponding columns

**Fig. 9:**
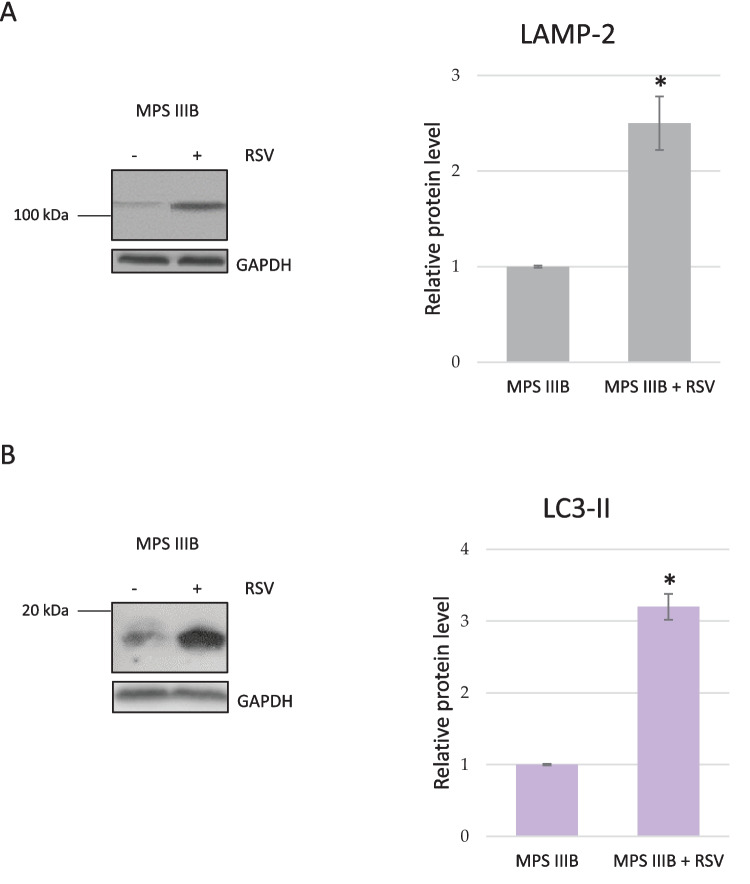
Levels of LAMP-2 (**A**) and LC3-II (**B**) proteins in the brains of MPS IIIB mice treated with water (minus (−) or MPS IIIB) or resveratrol at 50 mg/kg/day (plus (+) or MPS IIIB + RSV). Representative blots (obtained using the classical Western blotting procedure) are shown in the left panels, and the quantified results are presented in the right panels as the mean values ± SDs. Statistically significant differences obtained for each group (*n* = 6) are marked with (*), which indicates *p* < 0.05 vs. MPS IIIB (i.e., MPS IIIB with water)

To test whether resveratrol can induce autophagy, we determined the levels of protein markers during autophagy. When comparing relative concentrations of the p62 protein, significantly higher levels were found in the brains and livers of untreated MPS IIIB mice compared to those in to wild-type animals. No significant differences in Beclin-1 levels were detected (Fig. 8). These results indicated that autophagy was impaired in the MPS IIIB mice without a specific treatment. Administration of resveratrol at 50 mg/kg/day for 22 days resulted in a significant decrease in the p62 level in two tested organs of the MPS IIIB mice, demonstrating enhanced autophagy relative to that in the water-treated Sanfilippo mice (Fig. 8). This conclusion was corroborated by the significantly higher levels of LAMP-2 and LC3-II proteins in the brains of the MPS IIIB mice treated with resveratrol compared to those in the untreated animals (Fig. 9).

Previous studies indicated that resveratrol can stimulate autophagy through an mTOR-independent pathway (for a review, see ref. [27]). We found that PI3K levels were decreased, and the levels of mTOR kinase substrates (TFEB, S6K, and 4E-BP1) were either decreased or unchanged in the resveratrol-treated MPS IIIB mice compared to the water-treated MPS IIIB animals (Fig. 8). These results confirmed that in these animals, resveratrol-induced autophagy proceeded independent of the mTOR pathway.

## Discussion

Sanfilippo disease (MPS III) is a severe inherited metabolic disorder that causes devastating changes in the central nervous system and a significantly shortened lifespan (which is limited to approximately two decades) [1]. Although four different genetic defects leading to deficiency of different enzymes cause this disease, the general symptoms are similar in subtypes A, B, C, and D, but the course of the disease and level of severity may vary [2]. Many therapies developed for this disease have been tested, but despite remarkable successes in preclinical experiments, the clinical trials performed to date did not indicate sufficient efficacy to abolish or even alleviate most of the symptoms. These failure treatments included enzyme replacement therapy [16, 17], gene therapy [15], and substrate reduction therapy [18, 49]. Therefore, there is an urgent need to develop a method for the efficient treatment of MPS III patients. In this study, we tested previously proposed hypotheses [23, 27, 31, 40] suggesting that stimulation of the autophagy process, especially with natural compounds, might be beneficial in the treatment of Sanfilippo disease.

In this study, several natural (poly)phenols or other natural autophagy stimulators, also known for their antioxidative activities, were tested in both cell and animal models. As a positive control, genistein was used because it reduces the synthesis of GAG [43–45] and stimulates lysosomal biogenesis [47]. Genistein has been demonstrated by various groups to slow the production of GAG in cultured fibroblasts [43, 45], and some studies showed an increase in GAG synthesis in chondrocytes [50]. The discrepancy among studies has been suggested to be due to different effects of genistein on GAG levels in different types of cells [51]. Nevertheless, experiments with animal models of MPS II and MPS IIIB demonstrated the efficacy of this isoflavone in reducing GAG levels and attenuating abnormal behavior [46, 52], but these benefits have not been observed in MPS I mice [53]. In fact, several clinical experiments and trials have been conducted to test the efficacy of genistein in the treatment of MPS patients (different types), but contradictory results have been reported, indicating either significant attenuation of symptoms [54–57] or a lack of considerable clinical benefit [18, 58–60]. One might assume that these ostensibly discrepancies may be due to the various sources of genistein used (which may influence the bioavailability of this compound in humans), different patient age ranges (younger patients might respond better to treatment) or the different tests that were performed to assess drug efficacy (the results of psychological and cognitive tests for this disease are especially difficult to interpret). The multiple effects of genistein indicate how difficult it is to unequivocally estimate the efficacy of a drug for MPS. Therefore, extensive studies, both preclinical and clinical, are required to analyze each potential therapeutic method proposed to be treat this disease.

In the experiments presented in this report, we used known natural autophagy stimulators, capsaicin [29, 30], curcumin [31–33], resveratrol [34, 35], trehalose [36, 37], and calcitriol [39]. Cytotoxic effects were observed only at high concentrations of curcumin (30 and 80 μM) (Fig. 2), indicating the general safety of the investigated compounds in MPS III cells. Moreover, a significant decrease in GAG levels was observed after treatment of cells obtained from patients with one of each subtype of MPS III with each tested molecules (Fig. 3). The positive effects of trehalose on MPS IIIB mice, due to the induction of autophagy, had been previously demonstrated [22], and the results of our experiments with cell models of all Sanfilippo disease subtypes corroborated this discovery. Among the tested compounds, resveratrol exerted the greatest effects (Fig. 3), and we confirmed its efficacy in the specific reduction in HS levels (Fig. 4). Unaffected HS levels in control (HFDa) cells (Fig. 4) may indicate that GAG molecules present at normal concentrations did not undergo accelerated decay via autophagy. If this is the case, pathological GAG is degraded after autophagy stimulation. In fact, resveratrol-mediated activation of the impaired autophagy pathway were confirmed in experiments with MPS IIIB mouse models (Fig. 8), which were also used in experiments on the effects of this compound in vivo. Unexpectedly, we found that resveratrol normalized urinary GAG levels (Fig. 5) and attenuated all tested abnormal behaviors in the affected animals (Figs. 6 and 7). These results provide proof of principle for considering natural autophagy stimulators, especially resveratrol, potential drugs for Sanfilippo disease treatment. This possibility reveals a new direction for further studies focused on developing a therapeutic procedure based on the use of these compounds. In fact, resveratrol appears to possess properties that result in reduced GAG (including HS) levels in MPS cells and in mouse urine, and it also attenuated the abnormal behaviors of Sanfilippo subtype B mice. Therefore, it seems that the recently published proposal suggesting that resveratrol may be potential treatment of mucopolysaccharidosis (especially Sanfilippo disease) [40] is substantiated.

Notably, resveratrol has been previously proposed as a compound that is potentially useful in the treatment of other lysosomal storage diseases. In studies on neuronal ceroid lipofuscinosis (also called Batten disease), resveratrol was found to increase the NAD^+^/NADH ratio and levels of ATP, p-AMPK, PGC-1α, and SIRT1, whereas decreased levels of p-S6K1 were observed in cell and mouse models of this disease treated with this compound [61]. Interestingly, we observed a reduction in the regulatory factor p-S6K1 in the brains of MPS IIIB mice treated with resveratrol (Fig. 8). An increased lifespan was reported for mouse models of neuronal ceroid lipofuscinosis [61], and we demonstrated improved behavior in animals. Notably, it has been proposed that resveratrol may act on neuronal ceroid lipofuscinosis by improving adaptive energy metabolism [61]. We propose that activation of autophagy is the major mechanism of this compound in MPS III, although a possible influence on cell metabolism cannot be excluded. Other beneficial effects of resveratrol have been reported in for a lymphoblast model of Batten disease; however, the study suggested that the action of this compound action was due mainly to its antioxidant properties [62]. Furthermore, subsequent experiments on the mouse *Ppt1*-knockout model demonstrated a reduction in T_H_17 cells, IL-17A, and MMPs and an elevation in the levels of tight junction proteins after administration of resveratrol, and improved blood‒brain-barrier integrity was observed under these conditions [63]. Therefore, resveratrol likely functions via different mechanisms to attenuate various abnormal cellular functions in Batten disease and possibly in other lysosomal storage diseases.

Another interesting mechanism of action of resveratrol was reported for Pompe disease (a Type II glycogen storage disease). When a leaky splicing mutation in the *GAA* gene was the cause of this disease, treatment with resveratrol resulted in an increase in the level of normally spliced mRNA, but the mechanism of this phenomenon remains unknown [64].

Beneficial effects of resveratrol have also been reported for Gaucher disease, a lysosomal storage disorder caused by deficiency of glucocerebrosidase. In Gaucher disease patient-derived fibroblasts, treatment with resveratrol caused a reduction in the storage of glucosylceramides [65] and enhanced the viability of these cells [66]. These outcomes were correlated with decreased levels of the apoptotic factors AIF, Bax, and cleaved caspase-3. On the other hand, the concentrations of ACAT1, E3BP, and CS were increased in resveratrol-treated cells [65, 66]. Interestingly, the possible involvement of autophagy modulation induced by resveratrol has been suggested in studies on mutants in the *Drosophila GBA1* gene (encoding glucocerebrosidase) [67]. An analogous *Drosophila* model bearing mutations in the *CG2135* gene, thus resembling human MPS VII (Sly disease), was used in experiments that demonstrated reduced neuromuscular pathology and restored motor functions after resveratrol administration [68]. Various potential mechanisms of action of this compound have been proposed, including antioxidant, anti-inflammatory, cellular energy homeostasis-controlling, and autophagy-modulating activities, but the true mechanism(s) remains to be elucidated. Intriguingly, in a murine model of Krabbe disease (caused by the deficiency of galactosyl-ceramidase), rapamycin (a strong activator of autophagy), but not resveratrol, partially restored the levels of autophagy markers that had been dysregulated [69]. Interestingly, excessive accumulation of p62 has been observed in the neurons of mice with Krabbe disease [63], similar to an outcome observed in MPS IIIB mice in our study (Fig. 8). Notably, resveratrol treatment resulted in a decrease in p62 levels in our model but not in the previous study (Fig. 8 and ref. [69]). These results indicate that the action of resveratrol may be different in different lysosomal storage diseases. Moreover, a recently published article indicated that (in contrast to previous reports [27, 34, 35] and the results presented in this work (Fig. 8), demonstrating stimulation of autophagy via resveratrol mediated through mTOR-independent pathways) this compound activated the autophagy process mediated by protein phosphatase 2A-dependent dephosphorylation of TFEB, a transcription activator that enhances the expression of genes encoding lysosomal proteins, which are inactive when phosphorylated by mTOR [70]. Therefore, the mechanisms of action of resveratrol may be even more complex than those identified to date.

In our behavioral studies, we noted an additional interesting observation: the hyperactive phenotype was generally not associated with neurodegeneration. Indeed, when we detected hyperactivity in untreated MPS IIIB mice (at 10 weeks), mouse brain GAG levels were low. Thus, the motor hyperactivity observed in these mice was not necessarily due solely to GAG accumulation. Reports in the literature indicated that a number of histological changes identified long before GAG levels were found to be elevated in the CNS. MPS IIIB mice accumulate the ganglioside GM2 and, more importantly, the ganglioside GM3 in brain cells. The consequences of these accumulated gangliosides in lysosomes and the cell membrane lead to extensive and not yet fully understood neuropathological changes. Moreover, the characteristic vacuolization of lysosomes that contain aggregates and deposits was detected in the brains of MPS IIIB mice as young as 6 weeks of age, and this vacuolization increased with disease progression [71]. Further indicators of an ongoing disease process in the brain were changes detected at an early stage, such as microglial activation, astrocytosis, or an extensive and intense inflammatory process. These histopathological changes clearly correlate with the clinical picture, which consists of a shortened life expectancy, as well as a number of behavioral abnormalities, including aberrant anxiety responses and locomotion activity levels, which have been previously detected in an open field test [71]. Therefore, although in our experiments performed with 10-week-old MPS IIIB mice with brain GAG levels that did not exceed those observed in other groups, GAG likely accumulated in specific brain areas, leading to specific deviation from the typical behavioral pattern, and at an early stage of the disease, these increased GAG levels likely resulted in the described behavioral changes, including hyperactivity, episodes of immobility, and increased anxiety responses.

However, the results of other recent studies performed with a mouse model of MPS IIIA indicated that autistic-like behavior is caused by the increased proliferation of mesencephalic dopamine neurons not lysosomal dysfunction [72]. Notably, these outcomes are characteristic of children suffering from Sanfilippo disease [5, 73, 74]; hence, the mechanisms underlying to storage disorders are likely to be similar (or the same) in mice and humans. Detailed experiments with a mouse MPS IIIA model suggested that pathological modulation of dopamine activity can be a result of altered HS function, not elevated GAG levels [72]. This possible connection was corroborated by the results presented in our study with MPS IIIB mice. Hyperactivity and increased vertical movements seemed to mimic autistic behavior, and we observed these movements before observing significant accumulation of GAG. Therefore, these results may reflect the same or a similar mechanisms of neuropathology in the MPS IIIA and IIIB subtypes, which are not necessarily simple consequences of excessive GAG storage. Hence, changes in both the expression of many genes and the pathways of various cellular processes in MPS cells as reported recently [11–14, 75] might contribute to the pathomechanisms of Sanfilippo disease, which differ from the direct consequences of the accumulation of GAG (or, more precisely, that of HS).

Considering resveratrol as a potential drug for treating MPS III, a neurodegenerative disease, it is worth noting that this compound has been found to cross the blood‒brain barrier [76]. However, recent quantitative studies indicated that resveratrol crossing this barrier was relatively inefficient, at a few percentage points [77]. Therefore, various attempts to increase the efficiency of resveratrol delivery to the brain (such as those proposed recently [78, 79]) may be especially important to the development of a true therapy based on this compound.

Notably, considerable literature data have supported the ability of resveratrol not only to remove toxic protein aggregates in a variety of disease models but also to confer protection, as reviewed recently [40]. For example, resveratrol conferred protection against early neuronal dysfunction observed in a transgenic *Caenorhabditis elegans* model expressing mutant polyglutamine. Many studies have confirmed the high potential of resveratrol protecting striatal neurons from death and progressive neurodegeneration resulting from mutant polyglutamine accumulation in a mouse model of Huntington’s disease [40]. The neuroprotective properties of resveratrol have also been confirmed in Alzheimer’s disease models, where it has been shown to enhance memory processes, as well as increase cell survival, through the stimulation of SIRT1-, AMPK-, and mTOR-dependent pathways, leading to a reduction in amyloid levels [40]. Therefore, it is likely that resveratrol can exert similar effects on patients with MPS III.

## Conclusions

Natural dietary (poly)phenols and other natural autophagy stimulators, known also for their antioxidant activity, efficiently reduced the levels of GAG in fibroblasts derived from patients suffering from all subtypes of Sanfilippo disease. Resveratrol normalized urinary GAG levels and attenuated abnormal behaviors in MPS IIIB model mice, supporting further studies on this compound as a possible drug for treating Sanfilippo disease. Autophagy stimulation appeared to be a mechanism of action of resveratrol in ameliorating MPS IIIB pathology; however, other functions of this molecule (such as its antioxidative effects) cannot be excluded, especially when considering its multiple biochemical functions.

## Supplementary Information

Below is the link to the electronic supplementary material.

Supplementary file1 (PDF 2722 kb)

Supplementary file2 (PDF 2739 kb)

Supplementary file3 (PDF 2551 kb)

Supplementary file4 (PDF 2763 kb)

Supplementary file5 (PDF 2732 kb)

Supplementary file6 (PDF 2552 kb)
